# Vitamin E Can Ameliorate Oxidative Damage of Ovine Hepatocytes *In Vitro* by Regulating Genes Expression Associated with Apoptosis and Pyroptosis, but Not Ferroptosis

**DOI:** 10.3390/molecules26154520

**Published:** 2021-07-27

**Authors:** Luyang Jian, Ying Xue, Yuefeng Gao, Bo Wang, Yanghua Qu, Shuanghong Li, Heqiong Li, Zhen Li, Bing Wang, Hailing Luo

**Affiliations:** State Key Laboratory of Animal Nutrition, College of Animal Science and Technology, China Agricultural University, Beijing 100193, China; Jianluyang@cau.edu.cn (L.J.); 18801586516@163.com (Y.X.); nexhyf@163.com (Y.G.); wangboforehead@163.com (B.W.); quyanghua@cau.edu.cn (Y.Q.); S20203040606@cau.edu.cn (S.L.); joan374261795@163.com (H.L.); lizhen6394@126.com (Z.L.); wangb@cau.edu.cn (B.W.)

**Keywords:** vitamin E, oxidative stress, non-antioxidation, apoptosis, pyroptosis, ferroptosis

## Abstract

(1) Background: the current research was conducted to investigate the potential non-antioxidant roles of vitamin E in the protection of hepatocysts from oxidative damage. (2) Methods: primary sheep hepatocytes were cultured and exposed to 200, 400, 600, or 800 μmol/L hydrogen peroxide, while their viability was assessed using a CCK-8 kit. Then, cells were treated with 400 μmol/L hydrogen peroxide following a pretreatment with 50, 100, 200, 400, and 800 μmol/L vitamin E and their intracellular ROS levels were determined by means of the DCF-DA assay. RNA-seq, verified by qRT-PCR, was conducted thereafter: non-treated control (C1); cells treated with 400 μmol/L hydrogen peroxide (C2); and C2 plus a pretreatment with 100 μmol/L vitamin E (T1). (3) Results: the 200–800 μmol/L hydrogen peroxide caused significant cell death, while 50, 100, and 200 μmol/L vitamin E pretreatment significantly improved the survival rate of hepatocytes. ROS content in the cells pretreated with vitamin E was significantly lower than that in the control group and hydrogen-peroxide-treated group, especially in those pretreated with 100 μmol/L vitamin E. The differentially expressed genes (DEGs) concerning cell death involved in apoptosis (*RIPK1*, *TLR7*, *CASP8*, and *CASP8AP2*), pyroptosis (*NLRP3*, *IL-1β*, and *IRAK2*), and ferroptosis (*TFRC* and *PTGS2*). The abundances of *IL-1β*, *IRAK2*, *NLRP3*, *CASP8*, *CASP8AP2*, *RIPK1*, and *TLR7* were significantly increased in the C1 group and decreased in T1 group, while *TFRC and PTGS2* were increased in T1 group. (4) Conclusions: oxidative stress induced by hydrogen peroxide caused cellular damage and death in sheep hepatocytes. Pretreatment with vitamin E effectively reduced intracellular ROS levels and protected the hepatocytes from cell death by regulating gene expression associated with apoptosis (*RIPK1*, *TLR7*, *CASP8*, and *CASP8AP2)* and pyroptosis (*NLRP3*, *IL-1β*, and *IRAK2*), but not ferroptosis (*TFRC and PTGS2)*.

## 1. Introduction

Vitamin E was first described as a compound that was essential for the reproduction of rats [1] and was widely recognized as a lipid-soluble, chain-breaking antioxidant that prevents the cyclic propagation of lipid peroxidation [2,3]. It is now generally accepted that vitamin E, as a potent antioxidant, protects the organism against oxidative stress via the inhibition of propagation of reactive oxygen species (ROS) reactions. By means of its antioxidant properties, vitamin E is able to mitigate the harmful effects of oxidative stress on the male reproductive organ as well as spermatogenesis [4]. In addition, high levels of dietary vitamin E supplementation could significantly increase the expression (both at the mRNA and protein levels) of oxidative enzyme-related genes, such as Glutathione peroxidase 3 and Glutathione S-transferase alpha 1, through its non-antioxidant properties [5].

While it is well-known that vitamin E eliminates the harmful effects of oxidative stress, few studies have focused on its potential non-antioxidant function in cell protection systematically. Our results also suggested that vitamin E increases the expression of genes in the testes through its non-antioxidant properties. The addition of vitamin E to the diet could modify the expression of the reproductive hormone-related genes *GATA-4* and *STAR* in the testes of prepubertal sheep [6], and affected the level of cell growth-, cell differentiation-, and skeletal system-related transcripts in the testis [7,8]. Vitamin E deficiency could disrupt gene expression networks during Zebrafish development. Vitamin E deficient embryos experienced overall disruption to gene expression associated with gene transcription, carbohydrate and energy metabolism, intracellular signaling, and the formation of embryonic structures [9].

The liver is one of the most important organs in the body, where not only various metabolic processes (e.g., energy, lipid, ferric acid, uric acid metabolism) take place, but it also plays a central role in the detoxification of certain food-derived compounds and noxious substances [10]. The multiple physiological functions of this organ are mainly ensured by hepatocytes, which constitute more than 80% of the cells of the liver. As the center of metabolic function, hepatocytes have an enormous potential for generating ROS: anoxia, alcohol, liver disease, viruses, even nutritional imbalance can all lead to oxidative stress. The widespread notion about oxidative stress is that an excessive production of pro-oxidants or the exhaustion of the cellular anti-oxidant defense mechanism can lead to the oxidative damage of proteins, nucleic acids, carbohydrates, and lipids, and radical ROS is generally thought to play a major role in the process. High levels of ROS can lead to the impairment of cell structure, loss of function of biomolecules, and even cell death, all of which are associated with various liver diseases [11]. It was also reported that ROS may directly or indirectly participate in the initiation of apoptotic or necrotic cell death [12].

A key question is whether vitamin E can decrease hepatocyte mortality by affecting hepatic genes expression once cells are exposed to ROS. Thus, the aim of this study was to systematically analyze the non-antioxidant-related protective effects and the mechanism of vitamin E on sheep primary hepatocytes exposed to oxidative damage.

## 2. Results

### 2.1. Vitamin E Inhibits H_2_O_2_-Induced Cell Death in Primary Sheep Hepatocytes

**Figure 1:**
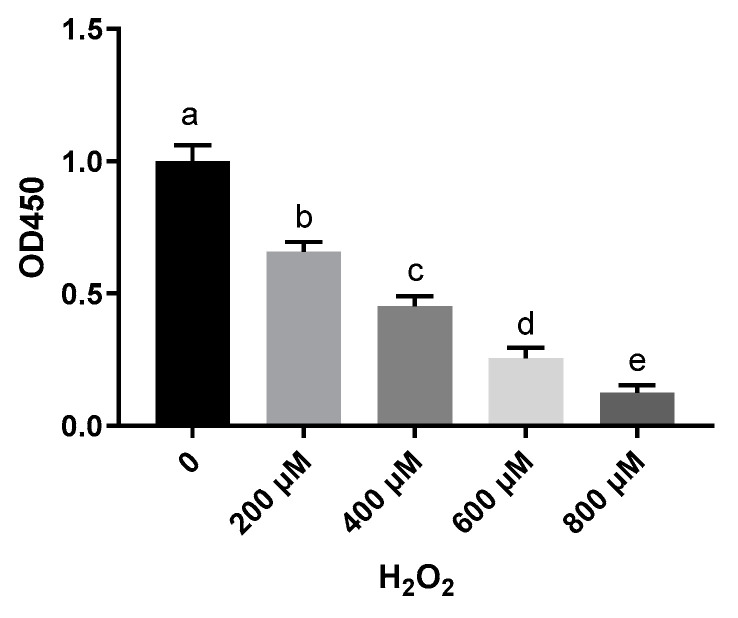
Hepatocytes’ survival rate detected by CCK-8 kit after being treated with 0, 200, 400, 600, and 800 μM H_2_O_2_. One-way ANOVA with Tukey’s test was applied. Data are expressed as mean ± SD; different letters indicate significant differences (*p* < 0.05).

**Figure 2:**
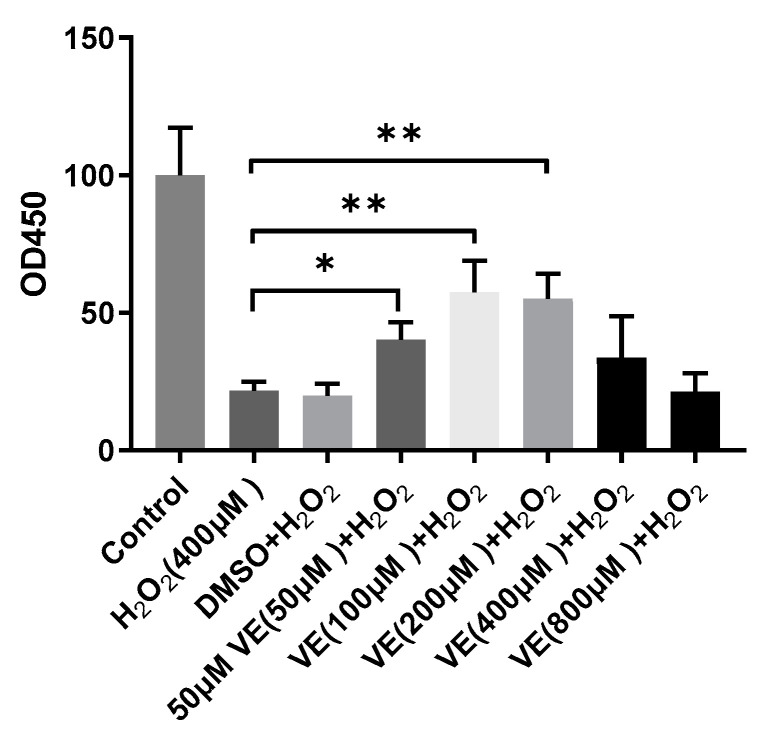
Hepatocytes’ survival rate detected by CCK-8 kit after being treated with 400 μM H_2_O_2_, with or without pretreatment of various concentrations of vitamin E(VE). One-way ANOVA with Tukey’s test was applied. Data are expressed as mean ± SD; the symbol * means *p < 0.05*, and ** means *p* < 0.01.

To investigate the effect of H_2_O_2_ on hepatocytes, cell viability was measured with a CCK-8 assay. Treatment of sheep hepatocytes with H_2_O_2_ at concentrations ranging from 200 to 800 μmol/L for 6 h led to a significant decrease in cell viability in a dose-dependent manner (Figure 1). Although H_2_O_2_ significantly reduced cell viability, a pretreatment with 50, 100, and 200 μmol/L VE effectively counterbalanced the negative effects of H_2_O_2_ and was able to prevent the decreases in cell viability caused by H_2_O_2_ (Figure 2). These results indicate that a prior treatment with VE protects primary cultured sheep hepatocytes from H_2_O_2_-induced cell death.

### 2.2. VE Ameliorates H_2_O_2_-Induced Oxidative Stress in Primary Sheep Hepatocytes

**Figure 3:**
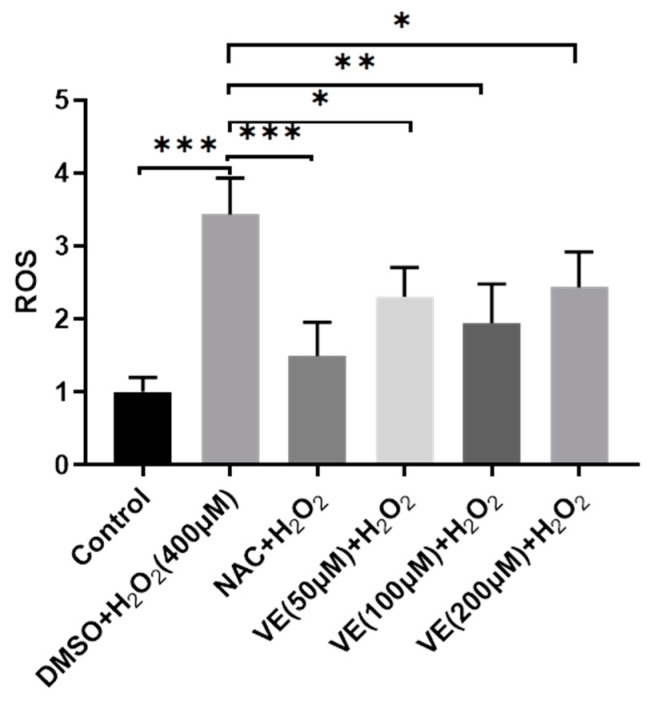
Hepatocytes’ survival rate detected by DCF-DA assay after being treated with 400 μM H_2_O_2_, with or without pretreatment of various concentrations of vitamin E or NAC. NAC was used as a positive control. One-way ANOVA with Tukey’s test was applied. Data are expressed as mean ± SD; the symbol * means *p* < 0.05, ** means *p* < 0.01, and *** means *p* < 0.001.

Compared with the control group, the addition of H_2_O_2_ to the culture medium significantly increased intracellular ROS levels in primary sheep hepatocytes cultured in vitro (Figure 3). However, this effect was significantly attenuated by a pretreatment with vitamin E, (50, 100, 200 μmol/L), or 1000 μmol/L N-Acetylcysteine (NAC, a known ROS scavenger, used as a positive control). Among the three vitamin E concentrations, 100 μmol/L vitamin E was most effective in reducing intracellular ROS levels.

### 2.3. Number of Genes and Differentially Expressed Genes

**Figure 4:**
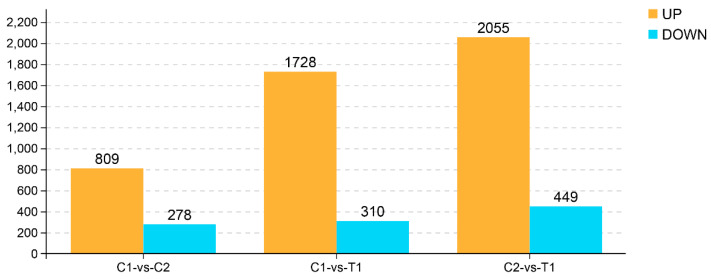
Number of significant DEGs among the three groups. The significant DEGs in the RNA-seq were identified following the rules of a false discovery rate (FDR) of < 0.05. C1 means control group, C2 means H_2_O_2_ treatment, and T1 means vitamin E and H_2_O_2_ treatment.

The fragments per kilobase of transcript per million mapped reads (FPKM) was used to determine the expression level of each gene. Differentially expressed genes (DEGs) were considered significant if the false discovery rate (FDR) was less than 0.05, and fold change was no less than 2. The number of significant DEGs among the three groups is shown in Figure 4. We identified 1087 significant DEGs between the C1 and C2 groups, of which 809 were upregulated and 278 were downregulated. A total of 2038 DEGs were identified between the C1 and T1 groups, and 1728 of these were upregulated and 310 were downregulated. Finally, a total of 2504 DEGs were identified between the C2 and T1 groups, 2055 of these were upregulated and 449 were downregulated.

### 2.4. GO Analysis of DEGs

**Figure 5:**
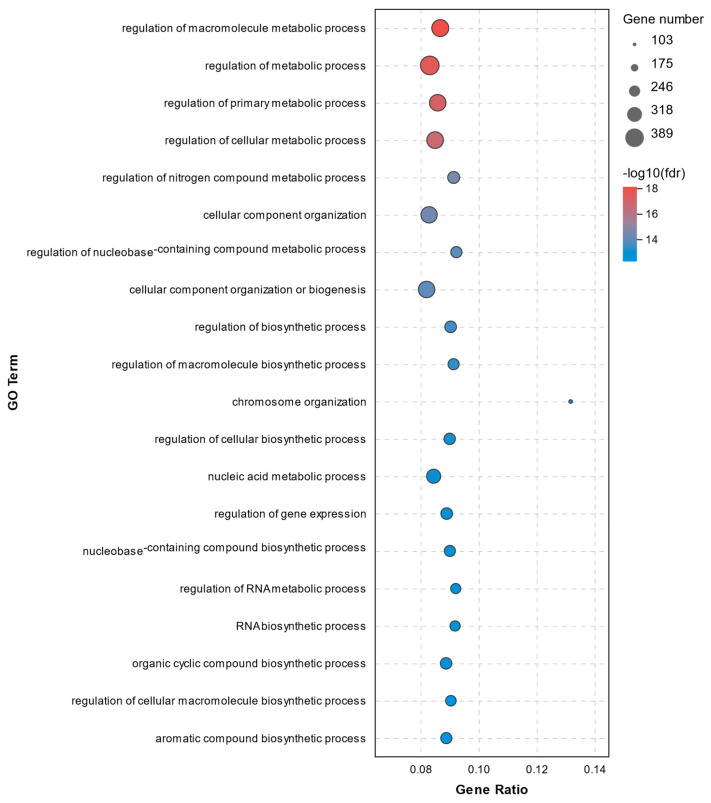
Top20 GO terms for DEGs between the C1 and C2 groups.

All DEGs were annotated according to three GO terms: biological process (BP), cellular component (CC), and molecular function (MF). A total of 494 significant enrichments between the C1 and C2 groups were observed with a *p* adjust-value of < 0.05 (Supplementary File S1). The top 20 GO terms for DEGs between the C1 and C2 groups are displayed in Figure 5. The data from the GO enrichment analysis indicated that the DEGs were mainly involved in metabolic processes and regulation, including regulation of macromolecule metabolic process, regulation of metabolic process, regulation of primary metabolic process, regulation of cellular metabolic process, and regulation of nitrogen compound metabolic process. Significant DEGs involved in these metabolic processes and regulation above were as follows: NLR family pyrin domain containing 3 (*NLRP3*, upregulated 12.15-fold), interleukin 1 beta (*IL-1β*, upregulated 2.41-fold), interleukin 1 receptor associated kinase 2 (*IRAK2*, upregulated 4.72-fold), caspase 8 (*CASP8*, upregulated 2.03-fold), caspase 8 associated protein 2 (*CASP8AP2*, upregulated 3.47-fold), receptor-interacting protein kinase 1 (*RIPK1*, upregulated 3.22-fold), toll like receptor 7 (*TLR7*, upregulated 5.50-fold), and prostaglandin-endoperoxide synthase 2 (*PTGS2*, upregulated 2.67-fold).

**Figure 6:**
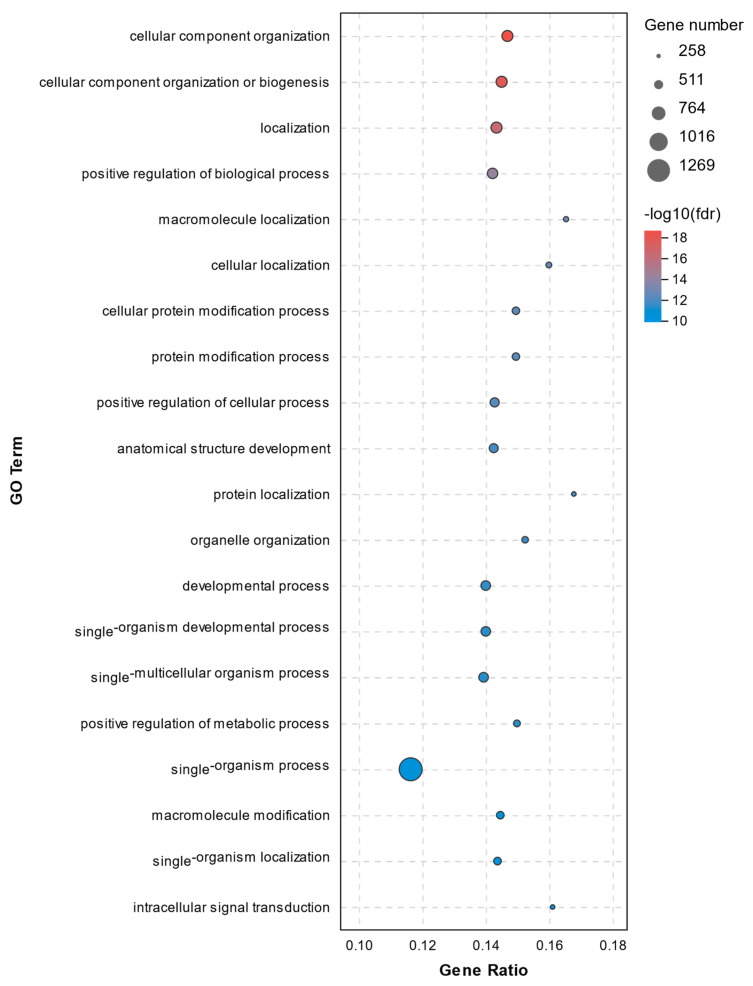
Top20 GO terms for DEGs between the C1 and T1 groups.

A total of 773 significant enrichments between the C1 and T1 groups were observed with a *p* adjust-value of < 0.05 (Supplementary File S2). The top 20 GO terms for DEGs between the C1 and T1 groups are shown in Figure 6. The GO analysis revealed that the cellular component organization, biogenesis and localization, and positive regulation of biological process were significantly enriched. Here, genes involved in processes above such as Prostaglandin-endoperoxide synthase 2 (*PTGS2*, upregulated 3.10-fold) and Transferrin receptor (*TFRC*, upregulated 7.96-fold) were worth mentioning. No significant differences were found in the abundance of *NLRP3*, *IL-1β*, *IRAK2*, *CASP8*, *CASP8AP2*, *RIPK1*, and *TLR7*.

### 2.5. KEGG Pathway Analysis of DEGs

**Figure 7:**
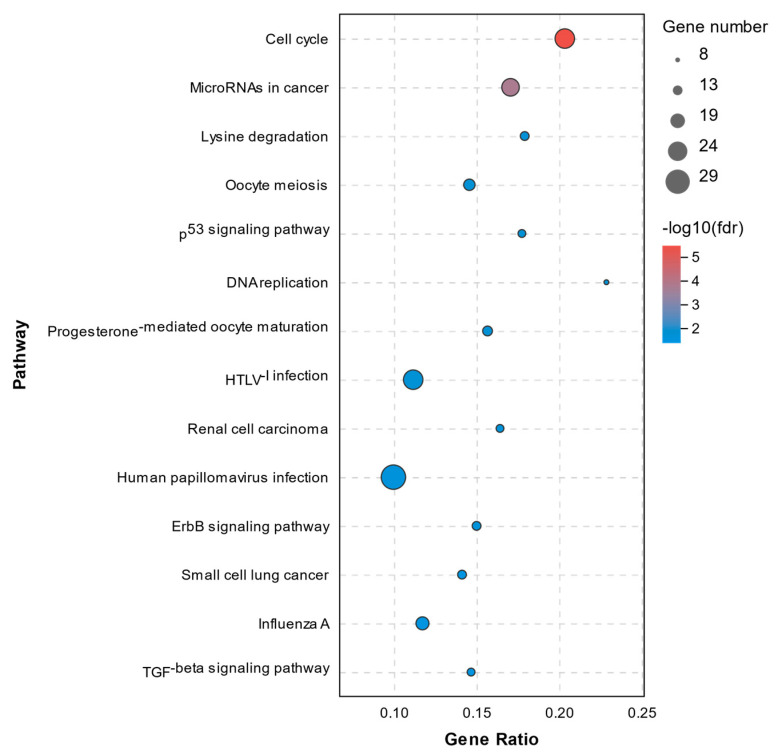
Top14 KEGG pathway analysis of DEGs between the C1 and C2 groups.

**Figure 8:**
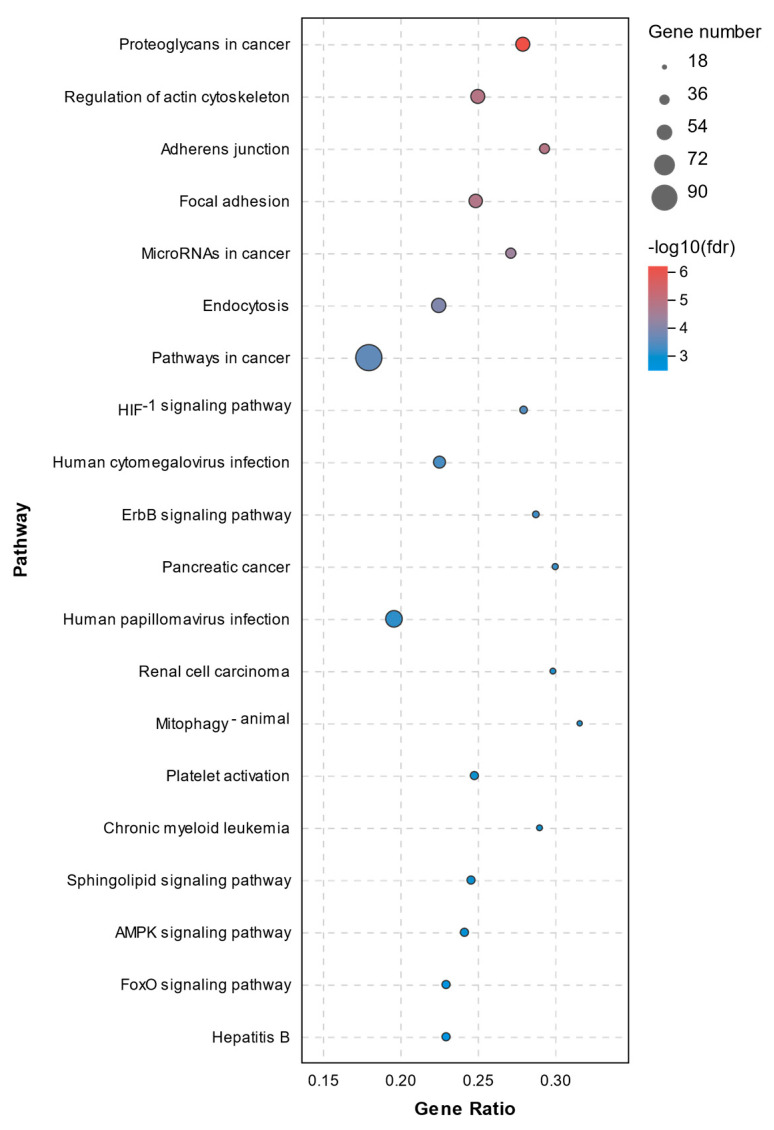
Top 20 KEGG pathway analysis of DEGs between the C1 and T1 groups.

The pathway annotation of DEGs was performed according to the KEGG database. All enriched pathways of DEGs in the C1 and C2 groups are shown in Supplementary File S3. The results demonstrated that there were 14 significantly enriched pathways with a *Q*-value of < 0.05, which are shown in Figure 7. The enriched pathways of DEGs between the C1 and T1 groups are shown in Supplementary File S4. There were 71 significantly enriched pathways with a *Q*-value < 0.05 (top20 pathways in Figure 8). None of them were closely related to cell death or cell mortality. The DEGs associated with cell death pathway, mentioned in GO analysis, were involved in apoptosis (*RIPK1*, *TLR7*, *CASP8*, and *CASP8AP2*), pyroptosis (*NLRP3*, *IL-1β*, and *IRAK2*), and ferroptosis (*TFRC* and *PTGS2*).

### 2.6. Validation of Genes by qRT-PCR

**Figure 9:**
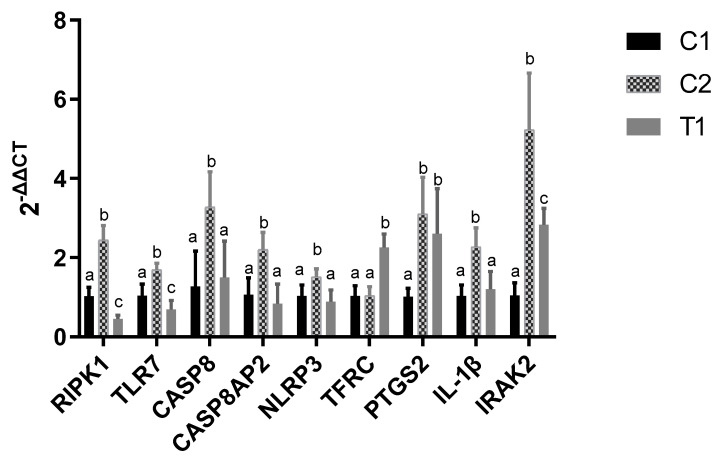
Relative expression of nine DEGs in the three groups. One-way ANOVA with Tukey’s test was applied. Data are expressed as mean ± SD. The same letter means no significant difference (*p* > 0.05), while different letters mean significant difference between groups (*p* < 0.05). C1 means control group, C2 means H_2_O_2_ treatment and T1 means vitamin E and H_2_O_2_ treatment. *RIPK1*, *Receptor-Interacting Protein Kinase 1; TLR7*, *Toll Like Receptor 7; CASP8*, *caspase 8; CASP8AP2*, *Caspase 8 Associated Protein 2; NLRP3*, *NLR family pyrin domain containing 3; TFRC*, *Transferrin Receptor; PTGS2*, *Prostaglandin-Endoperoxide Synthase 2; IL-1β*, *Interleukin 1 Beta; IRAK2*, *Interleukin 1 Receptor Associated Kinase 2*.

Nine DEGs were selected for further qRT-PCR analysis, including *NLRP3*, *IL-1β*, *IRAK2*, *CASP8*, *CASP8AP2*, *RIPK1*, *TLR7*, *PTGS2*, and *TFRC* (Figure 9). Compared with C1 group, we found that the qRT-PCR results were consistent with the RNA-seq data. Additionally, compared with H_2_O_2_ treatment (C2), relative expression of *RIPK1*, *TLR7*, *CASP8*, *CASP8AP2*, *NLRP3*, *IL-1β*, and *IRAK2* were down-regulated by pretreated vitamin E in the T1 group.

## 3. Discussion

When adequate redox homeostasis is compromised due to high amounts of ROS, the cell undergoes cell death [13]. In the current study, the addition of 200–800 μmol/L hydrogen peroxide led to the death of a large number of hepatocytes, whereas a 100 and 200 μmol/L vitamin E pretreatment could significantly improve cell survival (*p* < 0.05). The result of the DCFDA/H2DCFDA assay showed that the intracellular ROS content in the hydrogen-peroxide-treated group was significantly higher than that in the control, while vitamin E effectively reduced the ROS content in the hydrogen-peroxide-treated cells, especially at a concentration of 100 μmol/L (*p* < 0.01). Meanwhile, ROS content in 50 and 200 μmol/L vitamin E pretreated group was significantly lower than that in the control (*p* < 0.05). Thanks to the difference in its significance, 100 μmol/L vitamin E was chosen for further experiments.

We found it in GO analysis that the DEGs were mainly involved in metabolic processes and regulation, including the regulation of macromolecule metabolic process, regulation of metabolic process, regulation of primary metabolic process, regulation of cellular metabolic process, and regulation of nitrogen compound metabolic process. These DEGs were mainly related to ferroptosis, apoptosis, and pyroptosis. Specifically, the *RIPK1*, *TLR7*, *CASP8*, and *CASP8AP2* genes are involved in apoptosis, *TFRC* and *PTGS2* are involved in ferroptosis, while *IL-1β* and *IRAK2* are known to be associated with pyroptosis. The abundance of *IL-1β*, *IRAK2*, *NLRP3*, *CASP8*, *CASP8AP2*, *RIPK1*, and *TLR7* mRNA increased significantly in response to hydrogen peroxide (C1 group), while the increment was smaller in the T1 group, which had been pretreated with vitamin E, except for *TFRC* and *PTGS*2, which were not downregulated by the vitamin E pretreatment. The results of qRT-PCR were consistent with the RNA-seq data.

RIPK1 is a key mediator of cell death and inflammation. The activation of innate immune receptors, such as TLR, can also lead to the activation of RIPK1, and studies have shown that these stimuli can induce necrotic apoptosis in vitro. Suppression of RIPK1 kinase in cells can directly promote RIPK1-dependent apoptosis (RDA) upon stimulation by TNF-a [14,15,16,17]. Thus, the relative abundance of *RIPK1* and *TLR7* transcripts from the present study suggests that hydrogen-peroxide-induced cell death may be due to the increased expression of *RIPK1*. The increased expression of TLR7 further promoted the expression of *RIPK1*. Meanwhile, a vitamin E pretreatment effectively inhibited the expression of *RIPK1* and *TLR7*, which ameliorated the oxidative damage induced by hydrogen peroxide in a non-antioxidant manner.

Caspase-8 is the molecular switch for apoptosis, necroptosis, and pyroptosis. Extrinsic apoptosis relies on the formation of a death-inducing signaling complex (DISC), which is always composed of FADD and caspase-8. The binding of these two proteins will lead to the onset of exogenous apoptosis [18]. CASP8AP2 can act as a downstream mediator for CASP8-induced activation of NF-kappa-B, which is required for the activation of CASP8 in FAS-mediated apoptosis. Meanwhile, Caspase-8 is the initiator caspase of extrinsic apoptosis [19] and inhibits necroptosis mediated by RIPK3 and MLKL [18]. Caspase-8 was shown to cleave several proteins involved in the regulation of necroptosis, including RIPK1 and RIPK3. Four independent studies have now identified RIPK1 as a critical substrate that is cleaved by caspase-8 to prevent excessive cell death and inflammation [20,21,22,23]. The embryonic lethality of RIPK1 D325A/D325A mice could not be rescued by RIPK3 or MLKL deficiency but could be fully prevented by combined inhibition of FADD-caspase-8-mediated apoptosis and RIPK3-MLKL-mediated necroptosis [20,23,24], showing that caspase-8-dependent cleavage of RIPK1 prevents both apoptosis and necroptosis. When the expression of *CASP8AP2* and *Caspase8* was dramatically suppressed, apoptosis of cardiac stem cells was also inhibited [25]. In terms of cell viability, the increased expression of *CASP8* may lead to both positive and negative changes, but the final result may be to lower cell viability. Thus, combining with the results of cell viability and mRNA abundances of *CASP8* and *CASP8AP2*, it seems hydrogen peroxide stimulated the self-protection mechanism in hepatocytes and promoted the expression of *CASP8* and *CASP8AP2*, while a pretreatment with vitamin E effectively protected the cells from hydrogen-peroxide-induced apoptosis and eliminated the need for self-protection.

Pyroptosis is identified as inflammatory caspases (mainly caspase-1)-dependent programmed cell death, and is closely associated with the activation of the inflammasome [26]. A wide array of extracellular stimuli can drive pyroptosis. In the canonical model of pyroptosis, inflammasome sensor proteins, such as NLRP3, recognize cellular stressors, including those from bacteria, viruses, toxins, ATP, uric acid crystals, silica, and DAMPs. These stressors activate NLRP3 indirectly through potassium efflux, which leads to NEK7 binding NLRP3 to trigger its oligomerization. NLRP3 subsequently activates caspase-1 via the adaptor protein caspase recruitment domain (ASC). Caspase-1 processes and activates IL-1β and IL-18, and also cleaves GSDMD to release the membrane pore-forming GSDMD-N domain. GSDMD-N pores promote the release of activated IL-1β and IL-18 [27]. IRAK2 encodes the interleukin-1 receptor-associated kinase 2, one of two putative serine/threonine kinases that become associated with the interleukin-1 receptor (IL1R) upon stimulation. IRAK2 was reported to participate in the IL1-induced upregulation of NF-Κb [19]. We found that compared to the control (C1) group, the relative abundance of the *NLRP3*, *IL-1β*, *IRAK2* transcripts in the C2 group was 12.15, 2.41, 4.72-fold greater, respectively, while no significant difference was detected in cells of the T1 group. However, no significant difference was found in the expression of caspase-1 among the three groups. Thus, hydrogen peroxide-induced cell death may be due to the increased expression of *NLRP3*, *TLR7*, *IL-1β*, and *IRAK2*. The pretreatment with Vitamin E effectively inhibited the expression of *NLRP3*, *TLR7*, *IL-1β*, and *IRAK2*, which ameliorated the oxidative damage induced by hydrogen peroxide in a non-antioxidant manner.

Ferroptosis is a non-apoptotic regulated cell death that is dependent on iron and ROS. It is believed to occur through the lethal accumulation of lipid-based ROS when the glutathione (GSH)-dependent lipid peroxide repair systems are compromised [28,29]. Ferroptosis is triggered when the endogenous antioxidant status of the cell is compromised, leading to lipid ROS accumulation that is toxic and damaging to the membrane structure. Yes-associated protein 1 (YAP1), the main effector of the Hippo signaling pathway, has been reported to regulate the process of ferroptosis [30,31,32]. TFRC is further identified as the transcriptional target gene of YAP1 for the induction of ferroptosis [31,33]. Antagonizing this signaling axis allows the proto-oncogenic transcriptional co-activator YAP to promote ferroptosis by upregulating several ferroptosis modulators, including ACSL4 and TFRC [31]. Upregulation of *PTGS2* mRNA was a key feature of ferroptosis [34]. When *PTGS2* in HT-22 and Neuro-2a cell lines was downregulated, ferroptotic cell death was inhibited, too [35].

Vitamin E was shown to protect cells against ferroptotic death in vitro [36] and in vivo in *Gpx4*^−/−^ knockout mice [37]. Moreover, vitamin E deficiency was linked to the premature development of neurodegeneration, which is associated with ferroptosis [37,38]. The relative abundance of the *PTGS2* transcripts in C2 was more significant than C1 group (*p* < 0.05), which suggests that elevated expression of *PTGS2* may be relevant in the process of hydrogen peroxide-induced cell death. However, compared with C2 group, Vitamin E pretreatment did not affect the expression of *PTGS2* notably, but effectively increased the expression of *TFRC* in the research. The possible reason may be that TFRC, as a vesicle marker, participates in vitamin E transport in hepatocytes from endocytic vesicles around the nucleus to cell membrane. The addition of vitamin E may have a positive effect on upregulating its expression.

## 4. Materials and Methods

### 4.1. Materials

Liver tissue of Australian White sheep was purchased from Tianjin Aoqun Animal Husbandry Pty. Ltd. (Tianjin, China). A Cell Counting Kit-8 (CCK-8) was purchased from Dojindo Laboratories (CK04, DOJINDO, Kumamoto, Japan). Hydrogen peroxide (H_2_O_2_; catalog # 323381), collagen (catalog # C7661), and type IV collagenase (catalog # C5138) were obtained from Sigma-Aldrich, Inc (St. Louis, MO, USA). Reactive Oxygen Species Assay Kit (DCFH-DA) was purchased from Beyotime Biotechnology (S0033S, Shanghai, China). Williams’ E medium, Glutamax, Pen Strep, and B27 were acquired from Thermo Fisher Scientific (Waltham, MA, USA). Antibodies against Cytokeratin 18 (CK-18) were obtained from Abcam (Cambridge, MA, USA). Goat anti-rabbit IgG (FITC conjugated) antibody was supplied by Proteintech (Wuhan, China).

### 4.2. Isolation and Characterization of Primary Sheep Hepatocytes (PSH)

Hepatocytes were isolated from sheep liver tissue. A modified two-step collagenase perfusion procedure was used to isolate primary hepatocytes from the tissue [39,40]. Briefly, the liver tissue was perfused with PB (perfusion buffer: 20 mmol/L Hepes, Pen Strep, 5.33 mmol/L KCl, 0.44 mmol/L KH_2_PO_4_, 4.17 mmol/L NaHCO_3_, 137.93 mmol/L NaCl, 0.33 mmol/L Na_2_HPO_4_, 5.56 mmol/L glucose) to remove residual blood cells, followed by PBE (perfusion buffer plus 1.25 mmol/L EDTA) perfusion. The tissue was then perfused with PBC (perfusion buffer plus 1 mg/mL collagenase type IV, 5 mmol/L CaCl_2_; Gibco, USA). All buffer solutions were prewarmed to 37 °C prior to the isolation process. The solution containing the mixed cells and debris was passed through a cell strainer (pore size 40 μm; Falcon, CA, USA). Subsequently, the filtrate was centrifuged at 50× *g* for 3 min at 4 °C, then washed three times with Williams’ Medium E (Gibco), containing 10% fetal bovine serum (FBS). The isolated hepatocytes were then seeded on cell culture plates coated with collagen type I, at a density of 2.5 × 10^5^/cm^2^ in culture medium (Williams’ medium E containing B27 (50×), Glutamax (100×), and Pen Strep (100×) for 24 h at 37 °C in a humidified atmosphere (5% CO_2_ in air). Images of the isolated PSH are shown in Supplementary Figure S1.

Immunofluorescence was used to characterize the primary sheep hepatocytes. Briefly, cells were fixed with 4% paraformaldehyde for 15 min, and permeabilized with 0.1% TritonX-100 for 10 min. After blocking of the non-specific binding sites with goat serum for 1 h, the cells were incubated overnight at 4 °C with diluted monoclonal mouse anti-CK-18 antibody, and then incubated with diluted FITC-conjugated goat anti-mouse IgG (Wuhan Sanying, Wuhan, China) for 1 h. Cell nuclei were stained with DAPI (Solarbio, Beijing, China) for 15 min. Images of cells were captured using a Nikon Eclipse Ts2 fluorescence inverted microscope (Tokyo, Japan). Representative immunofluorescence images are shown in Supplementary Figure S2.

### 4.3. Cell Viability Assay

For the assay, sheep primary hepatocytes were cultured in 96-well plates (Corning, NY, USA) in six duplicates for each group. Cells in the control group received no specific treatment. Cells in the H_2_O_2_ group were treated with 200, 400, 600, or 800 μmol/L H_2_O_2_ and cultured at 37 °C for 6 h. For this purpose, 10 μL of CCK-8 solution were added to each well followed by further incubation at 37 °C for 3 h. Absorbance was measured at 450 nm using a microplate reader (MK3, Thermo Fisher Scientific, WA, USA). The values were converted from absolute counts to a percentage of the control. Finally, cells were pre-incubated with 50, 100, 200, 400, 800 μmol/L vitamin E for 12 h and then washed with PBS for 5 times before being treated with 400 μmol/L H_2_O_2_ for 6 h. Cells in the control group were treated with methyl sulfoxide, which was the solvent of vitamin E. Cell viability was measured using a CCK-8 assay kit, too. All values are expressed as the mean ± SD of six replicates.

### 4.4. Detection of Intracellular ROS

The DCFDA/H2DCFDA cellular ROS assay kit (Abcam; Cambridge, MA, USA) was used to assess intracellular ROS levels. Sheep primary hepatocytes cultured in 6-well plates (Corning, NY, USA) were treated with 400 μmol/L H_2_O_2_ with or without pre-incubation with vitamin E (50, 100, 200 μmol/L) or 1000 μmol/L N-Acetylcysteine (NAC, a known ROS scavenger, used as a positive control) for 6 h. The cells were then placed in the dark and incubated in the presence of 5 μmol/L DCF-DA for 30 min at 37 °C. Next, the cells were washed three times with phosphate-buffered saline (PBS) and measured at 535 nm following excitation at 485 nm using Tecan Infinite M200 Pro (Life Technologies, Thermo Fisher Scientific, Waltham, MA, USA).

### 4.5. Sample Preparation, RNA Extraction, Library Construction, and Sequencing

Sheep primary hepatocytes were cultured in 100 mm culture dishes (430167, Corning, NY, USA) until confluence. Cells in the C1 group received no specific treatment. Then, cells in the C2 group were treated with 400 μmol/L H_2_O_2_ for 6 h; while those in the T1 group were pre-incubated with 100 μmol/L vitamin E for 12 h followed by a treatment with 400 μmol/L H_2_O_2_ for 6 h. Each treatment was completed in triplicates. The samples were delivered to Gene Denovo Biotechnology Co. (Guangzhou, China) for RNA extraction, library construction, and sequencing. Total RNA was isolated using the Trizol reagent (Invitrogen, Waltham, MA, USA) according to the manufacturer’s instructions. The concentration and purity of the extracted RNA was determined with a NanoDrop 2000 spectrophotometer and Qubit 2.0 Fluorometer (Thermo Fisher Scientific, Waltham, MA, USA); the integrity of the RNA was evaluated using the Bioanalyzer 2100 system (Agilent Technologies, Santa Clara, CA, USA). Oligo (dT) beads were used for mRNA enrichment after total RNA extraction. The mRNA was then fragmented into pieces by fragmentation buffer and reverse-transcribed into first-strand cDNA using random primers, followed by second-strand cDNA synthesis by adding buffer, dNTP, DNA polymerase I, and RNase H. The cDNA was ligated to Illumina sequencing adapters after purification with the QiaQuick PCR extraction kit, end repair, and Poly(A) tail addition. The products were separated by size using agarose gel electrophoresis, amplified by PCR, and finally sequenced using Illumina HiSeqTM 4000 platform by Gene Denovo Biotechnology Co. (Guangzhou, China).

### 4.6. Quality Control and Alignment with a Reference Genome

First, raw reads were acquired from the sequencing platforms. This was followed by filtering, as adapters and low-quality bases that influence the process of assembly and analysis had to be removed. Ribosomal RNA (rRNA) was also removed by mapping reads to an rRNA database using Bowtie2 [41] to ensure the quality for further analysis. Clean reads of each sample were then aligned to the sheep reference genome (Oar_v4.0) by Tophat2 (version 2.0.3.12) [42]. The data were mapped according to the following principles: (a) maximum read mismatch is 2; (b) the distance between mate-pair reads is 50 bp; (c) the error of distance between mate-pair reads is ±80 bp.

### 4.7. Transcript Assembly and Gene Abundance Quantification

The alignments were used for the reconstruction of transcripts by means of the Cufflinks software package [43]. The program constructed faux reads based on the reference to remedy the influence of low-coverage sequencing. The reassembled fragments were mapped with reference genes and the identical ones were removed. After this, the Cuffmerge software was used to merge transcripts from each replica of one group into an integrated set of transcripts. The transcripts from the various treatment groups were then merged again into an integrated set of transcripts for further analysis.

Gene abundances were quantified by RSEM [44]. The level of gene expression was normalized by the FPKM (fragments per kilobase of transcript per million mapped reads) value. This calculation method can eliminate the effects of different gene lengths and amounts of sequencing data, which assures that the gene expression obtained can be immediately used for the analysis of differentially expressed genes (DEGs) among the samples. Raw RNA-seq data presented in this paper were submitted to the NCBI Short Read Archive (accession number: PRJNA738602).

### 4.8. Summary of the RNA-Seq Data

A total of 45836950, 36568200, 40,497,724 raw reads for the C1 group (C1-1, C1-2, C1-3), 36680694, 73870504, 40,981,816 raw reads for the C2 group (C2-1, C2-2, C2-3), and 54986892, 41448910, 40,095,894 raw reads for the T1 group (T1-1, T1-2, T1-3) were obtained in this study (Supplementary File S5).

### 4.9. Identification of DEGs

The DESeq2 package (http://www.rproject.org/, accessed on 30 June 2021) was used to analyze differentially expressed genes (DEGs) among the treatment groups. The significant DEGs were identified following the rule of a false discovery rate (FDR) of < 0.05. Gene Ontology (GO) and pathway enrichment analyses were then applied to the DEGs. First, all DEGs were aligned to GO terms in the Gene Ontology database (http://www.geneontology.org/, accessed on 30 June 2021). Gene numbers were then calculated for each term, and the significantly enriched terms were defined by the hypergeometric test. A *Q*-value of *0.05* was considered as a threshold. Pathway enrichment analysis was also performed based on the Kyoto Encyclopedia of Genes and Genomes (KEGG) database (Minoru et al., 2007). The calculation method was the same as that in the GO analysis. Those meeting the *Q*-value < 0.05 requirement were considered as significantly enriched pathways.

### 4.10. Validation of RNA-Seq Data

Nine DEGs were selected for quantitative real-time polymerase chain reaction (qRT-PCR) to validate the RNA-seq data. Three samples (each with four replicates) in each group were used for qRT-PCR. Total RNA was extracted from all these samples using the Trizol reagent. cDNA was then synthesized using a TRUEscript 1st Strand cDNA Synthesis Kit (with gDNase) according to the manufacturer’s instructions (Aidlab Biotechnologies Co., Ltd., Xiamen, China). The house-keeping gene β-actin was used as a reference. Primer sequences for the selected genes are given in Table 1. The qRT-PCR was performed at 95 °C for 15 min, followed by 40 cycles at 95 °C for 10 s and 60 °C for 30 s in an FQD-96A real-time PCR system (BIOER, Hangzhou, China) using Sybr Green qPCR Mix (Aidlab Biotechnologies Co., Ltd., Xiamen, China), and finished with the melting curve analysis. Relative gene expressions were calculated by the 2^−∆∆Ct^ method.

### 4.11. Statistical Analysis

Statistical analysis was performed by one-way ANOVA for comparing several groups using a General Linear Model in SPSS version 22.0 (SPSS, IBM, Inc., Chicago, IL, USA). All values were expressed as mean ± standard deviation (SD). Statistical significance was established at *p* < 0.05.

## 5. Conclusions

Oxidative stress induced by H_2_O_2_ caused hepatocyte damage and led to cell death. Vitamin E reduced intracellular ROS levels in the treated cells and decreased hepatocyte mortality through the suppression of *IL-1β*, *IRAK2*, and *NLRP3*, which were associated with apoptosis, and *CASP8*, *CASP8AP2*, *RIPK1*, and *TLR7*, associated with pyroptosis. Meanwhile, *TFRC* and *PTGS2*, associated with ferroptosis, were upregulated by vitamin E.

## Figures and Tables

**Table 1:** Details of primers used for qRT-PCR validation of RNA-seq.

Gene	Primers	Product Size (bp)
β-actin	5′ CATCGTCCACCGCAAAT 3′	17
	5′ GCCATGCCAATCTCATCTC 3′	19
NLRP3	5′ TGCAGCCTCACATCACA 3′	17
	5′ ATCACCCAGGTCGTTGTT 3′	18
IL-1β	5′ CAGCCGTGCAGTCAGTAA 3′	18
	5′ TGTGAGAGGAGGTGGAGAG 3′	19
IRAK2	5′ TCACCCATGTCCTGTCAA 3′	18
	5′ TGCCCCACTCTGATGAA 3′	17
CASP8	5′ AGGCAATGGTTTCACAGG 3′	18
	5′ TCCACCAGGCTTTTATGC 3′	18
CASP8AP2	5′ AAGAGGACGCATCTGAACA 3′	19
	5′ TACTGAAAGCCTGGAGCAA 3′	19
PTGS2	5′ CCAGGGAGACAATGCTTCT 3′	19
	5′ TGCAGCCTTAAACCCAGT 3′	18
TFRC	5′ AGAGTTTCCTTTCCGACACA 3′	20
	5′ CAGCTCCCTGAATAGTCCAA 3′	20
RIPK1	5′ TCTCGGGTTGTGTGTTTCC 3′	19
	5′ ACATGGCCGCTTTCCTT 3′	17
TLR7	5′ TCCATTTCCTTGCACACC 3′	18
	5′ CCATCTTTGGGGCACAT 3′	17

## Data Availability

Raw RNA-seq data presented in this paper were submitted to the NCBI Short Read Ar-chive (accession number: PRJNA738602).

## Supplementary Materials

The following are available online, Supplementary Figure S1: Image of primary sheep hepatocytes (24h after separated), Supplementary Figure S2: Purity of primary sheep hepatocytes detected by immunofluoresence, Supplementary File S1, Supplementary File S2, Supplementary File S3, Supplementary File S4, Supplementary File S5.

## Sample Availability

Not available.
