# Supplementary figures and images for: Vitamin E Can Ameliorate Oxidative Damage of Ovine Hepatocytes In Vitro by Regulating Genes Expression Associated with Apoptosis and Pyroptosis, but Not Ferroptosis
Source: Molecules. 2021 Jul 27;26(15):4520. doi: 10.3390/molecules26154520 (PMC8348559; doi:10.3390/molecules26154520)

## Supplementary figure S1

Image of primary sheep hepatocytes (24h after separated)

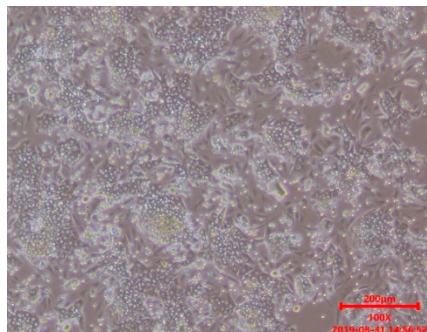

100X

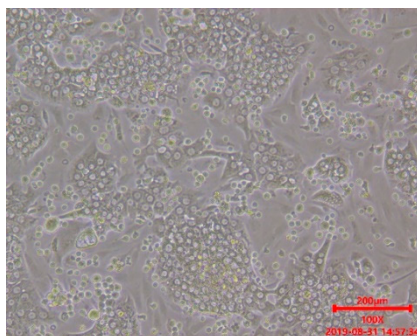

200X

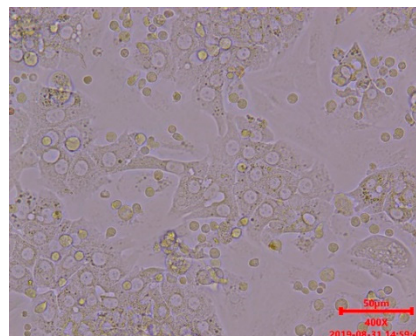

400X

Supplement: Supplementary file 1 [file molecules-26-04520-s001.zip › molecules-1301163-supplementary Figure S1.pdf]

**Supplementary figure S2**

Purity of primary sheep hepatocytes detected by immunofluorescence

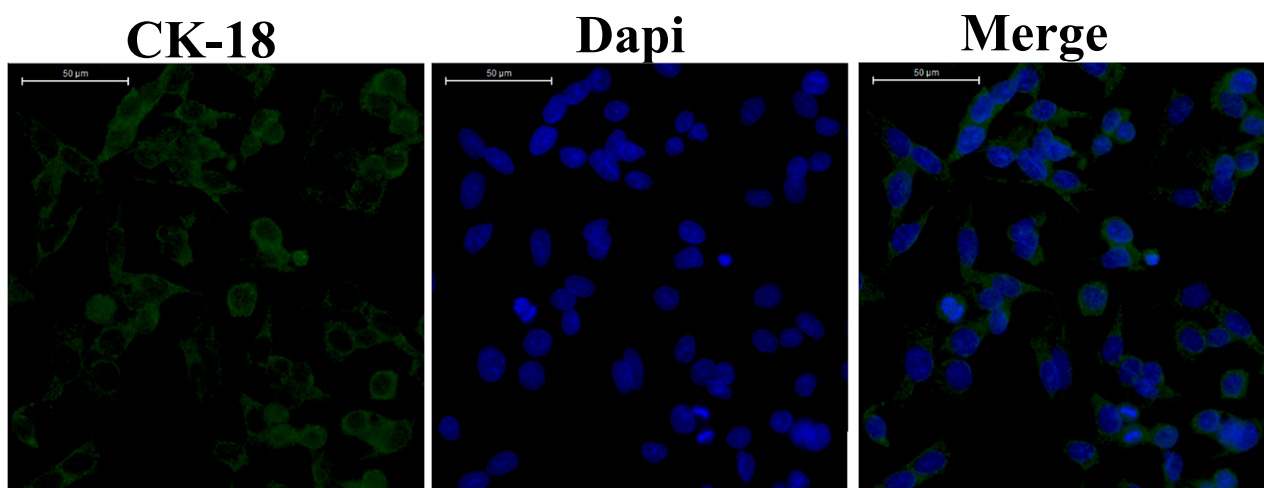

Supplement: Supplementary file 1 [file molecules-26-04520-s001.zip › molecules-1301163-supplementary Figure S2.pdf]
